# Trends in Research on Art Therapy Indexed in the Web of Science: A Bibliometric Analysis

**DOI:** 10.3389/fpsyg.2021.752026

**Published:** 2021-11-19

**Authors:** Natalia Rodriguez Novo, Maria Mercedes Novo Muñoz, Leticia Cuellar-Pompa, Jose Angel Rodriguez Gomez

**Affiliations:** ^1^Departamento de Enfermeria, Universidad de la Laguna, San Cristóbal de La Laguna, Spain; ^2^Instituto de Investigación en Cuidados del Colegio Oficial de Enfermeros de Santa Cruz de Tenerife, Santa Cruz de Tenerife, Spain

**Keywords:** art therapy, bibliometric, trend analysis, visual arts, health

## Abstract

**Aim:** Despite the increase in international research in art therapy, few studies have been developed with a bibliometric approach which describe the situation regarding this area of knowledge. Thus, the aim of this study is to describe and contextualize international scientific production in the visual arts modality in the context of artistic therapies, to offer a broader and more in-depth vision of the structure of this area of knowledge through of a bibliometric analysis of the publications indexed in the core collection of the Web of Science.

**Methods:** This is a retrospective, exploratory and descriptive, cross-sectional study to analyze the bibliographic data retrieved from the databases of the core collection. The analysis parameters included the data corresponding to the production according to type of document, country, journal, and institution. In addition, the main lines of research were located and classified and the subject matter of the most cited articles in each of them was summarized. Four periods were selected, between 1994 and 2020, to facilitate the thematic analysis and offer an evolutionary perspective of art therapy research.

**Results:** A total of 563 works were published, in 250 journals, in the 63 years between 1958, when the first document was published, and April 2021. The annual growth rate was 7.3% with a mean average of 8.7 publications per year, and 83.13% of the published works were articles. A total of 1,269 authors from 56 countries were counted. The mean number of citations per document was 5.6 and the mean number of citations per document and year was 0.6. The main research domains were psychology and/or rehabilitation and the highest production on this topic was concentrated in only three journals. In general, a high degree of variability was observed in the study topics and numerous theoretical and methodological articles. The most used visual arts modalities were in the main drawing, painting and photography.

**Conclusion:** This work did not find previous existence of any bibliometric analysis on the international scientific production in art therapy. In general terms, there has been a substantial growth in the number of publications on the subject over the last decade. However, this research area does not appear to have peaked, but, on the contrary, is still growing and progressing despite its long history in clinical practice.

## Introduction

As a result of international research in art therapies, they are increasingly being accepted as a health promoting practice (Stuckey and Nobel, 2010; Jensen and Bonde, 2018). Scientific evidence in this regard shows that these types of interventions positively influence both physical and psychological health, while improving social relationships (Stuckey and Nobel, 2010; Jensen and Bonde, 2018). In general terms, art therapy is a type of intervention that uses the creative process as a way to meet a therapeutic objective where different artistic disciplines can come together, as an instrument at the service of the healing process (Gacto and Gacto, 2012).

According to the Spanish Professional Association of Art Therapists, “Art therapy is a form of therapy that uses visual and artistic languages to facilitate the containment, exploration and resolution of conflicts. It is a healthcare profession, characterized by the use of artistic means and processes, to help contain and solve people’s emotional or psychological conflicts. In art therapy, the artistic creation process and the resulting objects act as intermediaries in the therapeutic relationship, allowing certain conflicting feelings or emotions to find complementary or alternative ways of expression to the word. The fields of application of art therapy extend to health, education and social assistance.”

On the other hand, the American Art Therapy Association (AATA) (American Art Therapy Association, 2021), defines art therapy as follows: “Art therapy provides the opportunity for non-verbal expression and communication, on the one hand, through involvement to solve emotional conflicts as well as to promote self-awareness and personal development.” It is about using art as a vehicle for psychotherapy, helping the individual to find a more compatible relationship between their inner and outer world.

Therefore, art therapy is understood to be a means by which an individual to know themselves, it requires an accompaniment of the person (in their process of inner growth) and a help for the person with social, educational, personal difficulties, through their artistic production, in such a way that the work carried out generates a process of transformation of the individual him or herself.

Different clinical guidelines of the National Institute for Health and Care Excellence (NICE) include art therapy as an indication with recommended evidence. Similarly, since the beginning of the 21st century, art therapy has been recognized in the Nursing Interventions Classification (NIC), as a Nursing intervention. Butcher et al. (2018), call it “Art Therapy” (4330), and it is defined as “the facilitation of communication through drawings or other forms of art.”

As complementary therapies, the different modalities of art therapy, among which are the visual arts, music therapy, dance therapy and drama therapy, are used to treat different psychological or cognitive behavioral disorders such as depression, stress, anxiety or neurological symptoms such as those caused by strokes (Wallace et al., 2004; Ozdemir and Akdemir, 2009; Hughes and da Silva, 2011; Eum and Yim, 2015; Sarid et al., 2017; Jang et al., 2018) or disorders derived from chronic diseases such as diabetes (Tang et al., 2021). These therapies provide benefits not only in the treatment or rehabilitation of the disease but also in the prevention of both certain disorders and the complications derived from them. Thus, different government agencies worldwide have drawn up evidence-based public policy documents that not only recognize the value of these interventions as alternative therapies, but also include their use in their recommendations (Galletly et al., 2016; Scottish Intercollegiate Guidelines Network, 2016; Sall et al., 2019).

In the specific case of the visual arts modality (drawing, illustration, painting, collage, photography and sculpture), art therapy not only prevails as a clinical intervention tool (Cheng et al., 2021; Hass-Cohen et al., 2021; Tang et al., 2021) but also as a diagnostic tool (Akhavan Tafti et al., 2021; Goldner et al., 2021; Grenimann Bauch and Bat Or, 2021), which gives it a versatile and innovative character, and which distinguishes it from the other art therapy modalities. Bearing in mind the above, and given the variety of studies and publications in this regard, it is of interest to have a more in-depth view of the scope and characteristics of the existing research; bibliometrics is the ideal instrument for this objective since by using bibliometric indicators, it is possible to evaluate scientific activity in any discipline or area of knowledge, and thus determine different basic categories that define who the producing people, institutions or countries are, how much they produce, what the impact of their publications is and how they collaborate with each other (Rodríguez Gutiérrez and Gómez Velasco, 2017).

After a thorough search of the literature, no research was found that involved a bibliometric study on scientific production in the visual arts modality within art therapy, therefore, this study could provide valuable information regarding the state of the question at hand in world research on the subject. Thus, the objective of the authors was to describe and contextualize international scientific production in the visual arts modality in the context of artistic therapies, to offer a broader and in-depth vision of the structure of this area of knowledge through a bibliometric analysis of the publications indexed in the core collection of the Web of Science (WoS), owned by Clarivate Analytics.

## Materials and Methods

### Design

This is a retrospective, exploratory and descriptive, cross-sectional study.

### Sample

Bibliographic data retrieved through a search strategy from databases including the core collection of the WoS were analyzed for the study. The data set included a total of 563 references.

### Data Source

After a selection process and subsequent decision-making, it was agreed that the WoS would be the most appropriate platform for data extraction, since it is a fundamental source of information for the evaluation of the research (Archambault et al., 2009). On the other hand, there is access to it, which from an operational point of view is essential to be able to obtain the necessary data. In addition, from a content point of view, its complete bibliographic data is available to develop a bibliometric analysis, and as such it is a widely used resource for this end (Archambault et al., 2009).

### Search Strategy

In order to define the search strategy, different preliminary tests were conducted using the advanced PubMed search, until a balance was achieved between the sensitivity and specificity of the results. The first step was to test the combination (art-therapy OR “Art Therapy”), once the first 100 results had been reviewed, it was concluded that it was necessary to add specific terms on visual arts to the strategy since the aim of the search was to identify publications on interventions with artistic therapies using visual techniques.

Finally, a search strategy was designed to obtain the corpus of information using the following combination of keywords: (art-therapy OR “Art Therapy”) AND (picture OR artwork OR illustrate OR photography OR painting OR paint OR “art galleries” OR “plastic arts” OR sculpture OR drawing OR draw).

Selection criterio:

Inclusion: as the present work is a type of analysis that aims to describe the state of the art in research in this domain, the objective of the search was to retrieve publications on the use of the visual arts as an art therapy technique, whether or not it is combined with another type of treatment, in any age group, within the health, educational or community sphere, without limits of year, country, etc.

Exclusion: any publication that does not refer to fine arts as an art therapy technique.

The search was conducted in the subject and title fields, with no date limit. The final execution date of the search was April 13, 2021.

### Data Extraction

As the database only allows the exportation of references in batches of 500, the results were downloaded twice and later, with the help of the Notepad++ for Windows text editing program, both downloads were merged into a single file for subsequent analysis. On the one hand, an Excel database was created in which the different fields of each record were categorized as follows: author names, title, source journal, abstract, organization, keywords, references, and page numbers.

### Analysis Methods

The analysis was performed by importing the data in txt format into an Excel spreadsheet using the function of obtaining external data. The analysis parameters included the data corresponding to the production by type of document, country, journal and institution. In addition, the main lines of research were identified and classified and the subject matter of the most cited articles in each of them was summarized.

The research lines were identified by dividing the corpus of analysis into different periods of 5 or 6 years, according to the publication dates of the articles. Keywords were identified and grouped by year, starting with 1994, the year when the first keywords were reported within the data retrieved for the analysis. After the periods had been defined, the main characteristics of the publications were identified through text mining in terms of the subject matter they address, the target population, the intervention environment, the pathologies treated and the type of art technique used.

### Validity, Reliability, and Rigor

Two researchers developed the selection process independently, with the aim of ruling out duplicate or incomplete references or those that did not exactly fit the study objective. In the case of differences, the investigators tried to reach an agreement with each other or requested arbitration from a third party.

## Results

### Descriptive Analysis

A total of 563 works were published, in 250 journals, in the 63 years between 1958, when the first document was published, and April 2021. The annual growth rate was 7.3% with a mean average of 8.7 publications per year and 83.13% of the published works were articles. The first article indexed in the reference database was published in the International Journal of Group Psychotherapy in 1958 (Potts, 1958). English with 507 (90.05%) publications was the main language of the publications, followed in second place by Spanish with 15 (2.66%), German was third with 14 (2.49%), and French was fourth with 11 (1.1%). A total of 1,269 authors from 56 countries were counted. The mean average number of citations per document was 5.6 and the mean average number of citations per document and year was 0.6. The description of the type of publication and search characteristics is shown in Table 1.

**TABLE 1:** Description, search characteristics, and results found in art therapy.

**Description**	**Results**
**Main information about data**	
Timespan	1958:2021
Sources	250
Documents	563
Mean average years from publication	8.72
Mean average citations per documents	5,595
Mean average citations per year per doc	0.5987
References	1
**Document types**	
Article	438
Article; book chapter	18
Article; early access	6
Article; proceedings paper	6
Book	1
Book review	12
Editorial material	6
Letter	2
Meeting abstract	8
Proceedings paper	38
Reprint	2
Review	26
**Document contents**	
Keywords Plus (ID)	687
Author’s Keywords (DE)	1432
**Authors**	
Authors	1269
Author appearances	1495
Authors of single-authored documents	181
Authors of multi-authored documents	1088
**Authors collaboration**	
Single-authored documents	198
Documents per author	0.444
Authors per document	2.25
Co-authors per documents	2.66
Collaboration index	2.98

### Evolution Over Time

**FIGURE 1:**
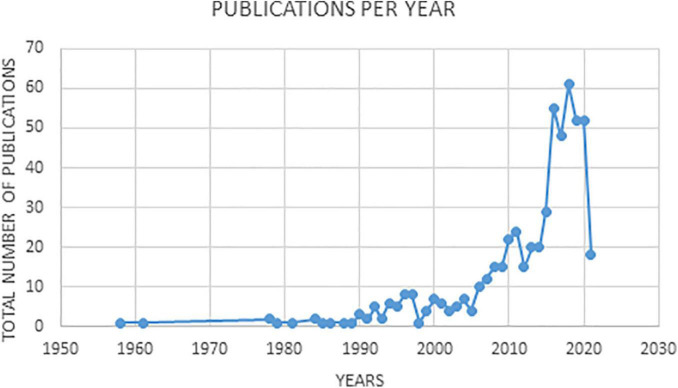
Evolution of the frequency of publications in art therapy over time.

Between 1958, when the first work was published, and 1977, only two articles were published. As of 1978, production fluctuated which lasted until 2005, when a clear upward trend began (Quinn et al., 2013), which accelerated in 2012. The most productive year was 2018 when 61 papers were published. The evolution of the publications can be seen in Figure 1.

### Distribution by Countries

An analysis was conducted by countries regarding the state of publications on art therapy. The results showed that, in the 63-year time window, from 1958 to 2021, authors from 53 countries participated with the United States, with 316 publications, leading the ranking of the most productive countries, followed by Israel (99) and the United Kingdom (69), in second and third place, respectively. Table 2 shows the countries with a minimum of 10 publications on the subject of study. In general terms, the United States was at the forefront in art therapy research, not only in terms of the volume of publications but also in terms of the number of citations received.

**TABLE 2:** Scientific production in art therapy, by country, with a minimum frequency of 10 publications.

**Country**	**Frequency**	**Total citations**	**Mean average article citations**
United States	316	1145	6,939
Israel	99	255	5,543
United Kingdom	69	287	7,972
Germany	66	224	9,333
Australia	55	174	6,692
China	55	88	3,826
Korea	45	147	6,682
Netherlands	40	24	2.4
Spain	27	10	0.833
Iran	26	14	1,556
Sweden	25	96	13,714
Canada	25	34	3.4
Italy	24	85	6,538
France	17	6	0.667
Poland	16	17	2,429
Russia	15	1	0.167
Japan	14	60	7.5
Turkey	14	15	3
Denmark	12	53	8,833
Serbia	10	14	2.8

### Distribution by Journals

Regarding the 250 journals that published works on art therapy, only three, belonging to the psychology and/or rehabilitation research domains, concentrated the largest production on this topic. Table 3 shows detailed information on the most productive journals in Art therapy.

**TABLE 3:** Journals with the highest production in art therapy publications.

**Main sources**	*NP	**Country**	**Research domain**	**TC	**h_index**	***IF JCR 2019	****Q
Arts in psychotherapy	132	England	Psychology	1047	17	1,322	Q3
			Rehabilitation				
Art therapy	40	United States	Rehabilitation	114	6	–	–
Frontiers in Psychology	24	Switzerland	Psychology	43	4	2,067	Q2

*NP, number of publications; **TC, total citations; *** IF JCR, impact factor in the journal citation report; ****Q, quartile.

**FIGURE 2:**
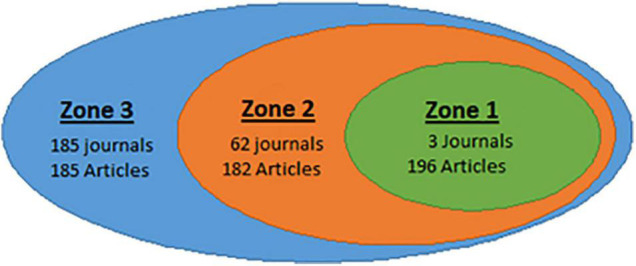
Scatter diagram of journals and articles on art therapy retrieved through the core WoS collection. Total number of journals: 250, total number of articles: 563.

Figure 2 shows a scatter diagram illustrating the quantitative relationship between the 250 journals and the 563 scientific articles analyzed in the study.

### Distribution by Institutions

**FIGURE 3:**
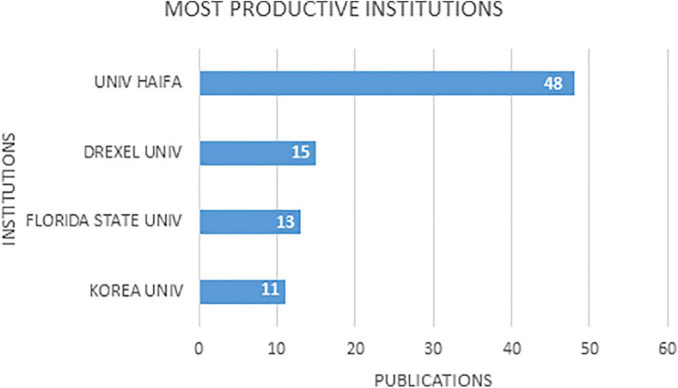
Most productive institutions in art therapy, based on the data analyzed.

In total, 639 institutions were recorded, which were classified into three performance levels. Of these, 466 (72.8%) were small producers, with only one published work, 169 (26.41%) were medium producers, with between two and ten works, and only 0.63%, with more than 10 works, were large producers. Of the four most productive institutions, two were from the United States, one from Israel, and one from Korea. Figure 3 is a graph with information on the four most productive institutions.

### Distribution by Authors

A total of 1,269 authors were counted, of which 181 corresponded to authors of single-author documents and 1,088 were authors of multiple-author documents. Regarding all the analyzed documents, 1,495 signatures were classified with a range of signatures per document from 1 to 12 signatures. The most productive author with nine papers was Seong-in Kim, from Korea University in Seoul, South Korea. Table 4 shows the authors with the highest scientific production in art therapy.

**TABLE 4:** Authors and frequency of publications on art therapy.

**Author**	NP*	**h_index**	TC**
Kim SI	9	6	80
Regev D	7	3	18
Snir S	7	3	15
Huss E	6	4	37
Kaimal G	6	3	25
Hanes MJ	5	3	20
Lev-Wiesel R	5	3	14
Or MB	5	3	34
Van Hooren S	5	3	16
Von Wietersheim J	5	3	17

*NP, number of publications; **TC, total citations.

**FIGURE 4:**
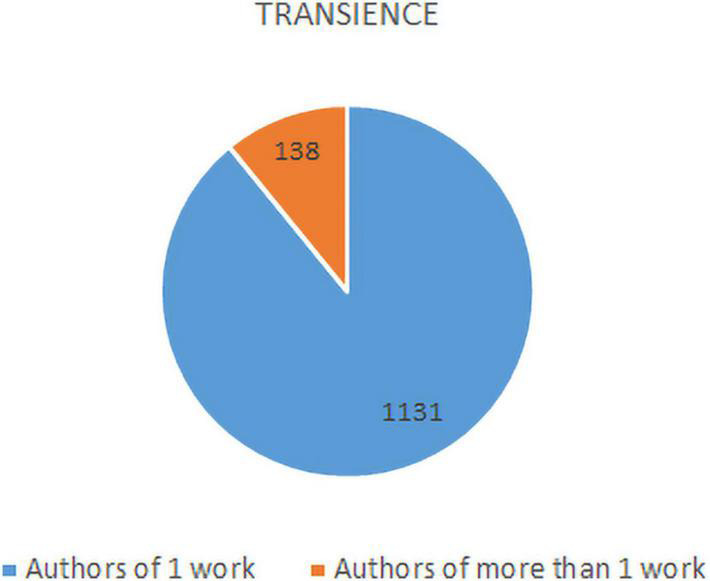
Transience of authors in art therapy publications.

As can be seen in Figure 4, almost 90% of the authors are transient authors, with only one publication.

Of the nine papers with a minimum of 50 citations, four were published in psychology journals, three in biomedical journals, and two in interdisciplinary journals. The journals The Arts in Psychotherapy and Psycho-oncology, monopolized 44.44% of the most cited works, with two articles each. Regarding the topic discussed, it was found that four of the most cited works were related to oncological issues, three to mental health, one related to nursing and another to gender violence. Table 5 shows the information related to the authors, topics covered, and citations.

**TABLE 5:** Reference of authors, topics covered, and citation of articles related to art therapy.

**References**	**Summary**	**Total citations**	**Mean average per year**
Bar-Sela et al., 2007	The aim of this study was to determine whether the improvement in depression, anxiety, or fatigue during chemotherapy after an anthroposophic art therapy intervention was sufficient to warrant a controlled trial.	86	5.73
Riley and Manias, 2004	The objective of this article was, through a bibliographic search, to provide a “snapshot” of the scope and uses of photography in clinical nursing practice and research.	84	4.67
Wood et al., 2011	The aim of this research work was to evaluate and synthesize the scientific evidence available for the use of art therapy in the management of symptoms in adults with cancer.	83	7.55
Geue et al., 2010	This article describes the content, concept and structure of art therapy interventions based on painting or drawing, as well as some additional methodical procedures and research results of art therapy in the field of psychosociology.	75	6.25
Favara-Scacco et al., 2001	Our goal is to prevent anxiety and fear during painful interventions, as well as prolonged emotional distress, through art therapy sessions for children with leukemia.	68	3.24
Parr, 2006	This article, based on qualitative data from in-depth interviews with artists from two Scottish mental health art projects, explores whether people with severe and long-lasting mental health problems experience belonging through their participation in a range of practices and contemporary artistic spaces.	64	4
Frohmann, 2005	This is a Scoping Review of the literature on community-centered support interventions with the aim of identifying and evaluating mental health outcomes among survivors of Intimate Partner Violence.	59	3.47
Cohen et al., 1988	Exploratory study in which a series of diagnostic drawings (DDS) was administered to 239 psychiatric hospitalized patients with a diagnosis of dysthymia, depression or schizophrenia with the objective of evaluating the link between the patients’ disease based on the psychiatric diagnoses and how it is expressed in the drawings.	52	1.53
Talwar, 2007	This article, following an overview of the role of memory and emotions in trauma and theories of art creation and brain function, proposes an art therapy protocol, designed to address the non-verbal core of traumatic memory, which has been successful in integrating the cognitive, emotional and physiological levels of trauma.	51	3.4

### Distribution by Research Areas

Of the 81 research areas in which works have been published on the art therapy modality that is being analyzed here, clinical psychology and rehabilitation are noteworthy as they account for 70.2% of all publications on the subject. Table 6 shows the results of the areas with a minimum of ten publications.

**TABLE 6:** Publications in art therapy, by research area.

**Ranking**	**Research area**	Records *	**% of 563**
1	Psychology clinical	211	37.48
2	Rehabilitation	184	32.68
3	Psychology multidisciplinary	73	12.97
4	Psychiatry	52	9.24
5	Psychology	25	4.44
6	Education educational research	23	4.1
7	Social sciences interdisciplinary	19	3.38
8	Health care sciences services	17	3.02
9	Nursing	17	3.02
10	Oncology	16	2.84
11	Public environmental occupational health	16	2.84
12	Integrative complementary medicine	14	2.49
13	Medicine general internal	14	2.49
14	Humanities multidisciplinary	12	2.13
15	Art	10	1.78
16	Social sciences biomedical	10	1.78

*The same work may correspond to more than one research area.

### Analysis of Keywords

In order to facilitate analysis and provide an evolutionary perspective of art therapy research, four periods were used: 1994–1999; 2000–2005; 2006–2010; 2011–2020. The year 2021 was not included because it is ongoing, and it was decided to report only the full years.

#### Period 1: 1994–1999

There was talk of creative arts therapies in this period and a high degree of variability is observed in the study topics, ranging from psychiatric disorders, dementia, feminism, speech disorders, palliative care, sexual abuse, disability, grief or infant migraine. Although a defined line of research was not observed, it could be said that the interventions in art therapy which took place in these early years were mainly performed in a hospital or institutional environment, with children and adolescents being the population mostly selected for this type of therapy. The techniques used also vary from drawing, collage, crafts to photography.

#### Period 2: 2000–2005

There is still a high degree of thematic variability. The first works on interventions in cancer patients appear in this period, as well as several publications on art therapy experiences aimed at healthy people, concerning family relationships, adopted adolescents, pregnant women and battered women. Similarly, intervention experiences in behavioral disorders, learning difficulties, victims of terrorist attacks, epilepsy, grief and psychiatric disorders such as schizophrenia and nervous anorexia and bulimia are addressed. The first publications appear here addressing the use of art therapy as a diagnostic tool, as well as extra-hospital interventions, developed on an outpatient basis in health, community or educational institutions or centers. Although a wide variety of experiences with children and adolescents is published, the adult population takes center stage. As for the techniques, the visual arts are mainly those belonging to modalities such as painting, drawing, photography, etc., but there are also descriptions of experiences of combined therapies integrating two or more types of artistic and psychological therapies such as art therapy, dance therapy, music therapy, and psychotherapy.

#### Period 3: 2006–2010

Numerous theoretical and methodological articles. Informatics and virtual work environments are introduced, along with digital video as a means of art therapy. Interest in the perspective of the therapist is appreciated, given the impact that their personal experiences in the therapeutic process can have on the results of the therapy. Similarly, vocational training is addressed. The publication of works on patients with serious psychiatric disorders such as schizophrenia and on cancer patients continues. Furthermore, psychopathologies such as trauma and post-traumatic stress in war veterans and in children and child sexual abuse are also addressed. Experiences on art therapy interventions in patients with chronic diseases such as HIV/AIDS, asthma and diabetes are published. Cognitive and behavioral strategies such as coping are worked on, emotional needs are addressed in frail elderly people as well as creativity in patients with dementia. Interest is maintained on the diagnostic use of art therapy through the creation and validation of evaluation tools. Outpatient, community and institutional interventions continue, including intervention experiences with prisoners. The different modalities of artistic therapies are combined with each other through the use of visual arts in all its modalities in combination with dance, music or narration, and alliances are generated with other types of therapies such as occupational therapy, cognitive therapies -behavioral, exercise, other types of complementary or alternative therapies together with healthy life strategies. There is a special interest in this period in research on the use of color, as an important factor in art therapy evaluations.

#### Period 4: 2011–2020

The use of computers, art materials in digital format, virtual environments and art applications for iPads and mobiles becomes consolidated in this period. Most of the interventions are aimed at adults. Thematic variety is observed in the retrieved results. The topics that generated the most attention were behavioral, psychological and communicative symptoms in patients with dementia, as well as the management of psychosocial difficulties such as fatigue, depression, anxiety and existential and relational concerns in cancer patients. Research continues into serious mental illnesses such as schizophrenia. To a lesser extent, work was published on intellectual disability, special educational needs, improvement of memory and thinking skills to achieve school competencies, socio-emotional problems in adolescents or motivation in children at risk of poverty. There was research on domestic violence and feminism, on epilepsy, childhood trauma, the constructive coping of children who go through experiences of forced relocation due to border changes, wars or natural disasters. Other topics discussed are related to depression, psychosis, as well as the identification of types of attachment in healthy people, as well as intervention in prisoners. In addition, studies aimed at children and adolescents focused on determining the efficacy of art therapy in relation to victims of sexual abuse, orphans, symptoms of separation, anxiety disorder or social adaptation and integration. Cognitive impairment in alcoholics and the development of the identity of the LGTBI community are hardly addressed. The efficacy of the different modalities of combined artistic therapies is addressed and special interest is given to the use of computers in the field of evaluation through art therapy. In general, the modalities of visual arts are drawing, painting, photography, modeling, construction with non-conventional materials, various multimedia techniques, and animation.

## Discussion

The analysis of scientific production, based on bibliometric parameters, is an essential tool for evaluating knowledge and determining the progress of disciplines and their fields. In the present case, given the specificity of the subject, this type of work provides useful information to understand the general properties of the state of knowledge about art therapy (Capilla-Díaz et al., 2020), since it offers a retrospective vision of the scientific production in the world based on the main bibliometric indicators.

Taking into account the annual progression of publications, a particular dynamic is observed with some periods of growth, alternating with others of regression. However, the increase in production from 2012 onward has shown a clear growth trend. This indicates that the topic has been gradually attracting the attention of professionals and researchers.

Regarding the geographical distribution of the publications, the results of the study are equivalent to those of a bibliometric study that analyzed the last 20 years of publications on music therapy (Li et al., 2021) since the 10 countries or regions that these authors identified as the main ones are among the twenty top producing countries in art therapy, with the United States heading the list in both cases.

A high degree of dispersion is observed at the journal level, fulfilling Bradford’s Law, since the scientific production in art therapy presents a highly unequal distribution given that a large number of articles are concentrated in a small population of journals, while the rest of the articles are scattered over a large number of journals. On the other hand, it seems logical that the main sources in this area, Arts in Psychotherapy, Frontiers in Psychology and Art Therapy, would be the journals chosen by the authors to publish their research, considering that they are specialized journals in art therapy. In the case of Frontiers in Psychology, although it is not specific to this area of research, it does belong to the field of psychology, one of the two domains identified in the present study as being main domains. This result is also similar to that of Li et al. (2021), mentioned above, where the authors found that the leading journals in the field were specialized in music therapy and where one of the main domains was also psychology.

Although two of the four most productive institutions were from the United States, it should be noted that the leading institution in this field is an Israeli university. Most of the institutions had a low performance in the scientific production on art therapy.

The most cited articles have a main interest in addressing symptoms with a psychological component such as anxiety, fatigue, emotional anguish or fear, during the course of serious diseases such as cancer, as well as psychiatric diseases such as schizophrenia or depression. In general, there is a clear line of research that refers to mental health. In addition, the different scientific domains present different citation practices, which for reasons of normalization of the bibliometric indicators are not comparable (González and González, 2016). Therefore, based on the results of this analysis it seems that, in general terms, the research in art therapy may not have an impact maturation speed of more than 5 years. This is probably due to the fact that as an area of research, it is still being development.

Regarding authorship, the most notable result is the high transience of the authors, which also points to the low level of development in research in the area of art therapy.

Art therapy is applied to a wide spectrum of health problems, through a wide variety of artistic modalities. In the last decade of the twentieth century, interventions took place in hospital and institutional settings, in hospitalized or inpatients, and according to the studies analyzed, they were mainly aimed at children. The first outpatient experiences in art therapy appeared in 2000, when it started diversifying out of the health field into educational settings and began to be used as a preventive therapy, applied to healthy people (Robertson, 2001; Swan-Foster et al., 2003), and for diagnostic purposes, with drawing or photography being incorporated into the assessment instruments (Hays and Lyons, 1981; Kim et al., 2011; Darewych, 2013). Experiences of combined therapies integrating two or more types of artistic therapies into psychological and/or pharmacological therapy have been described. As of 2006, with the development of information technology and the internet, the virtual environment and tools in digital format started to gain momentum as a means of artistic therapy. This development in art therapy has been growing stronger since 2010, with the improvement of mobile terminals, applications for digital tablets and mobile phones.

There are no established research lines concerning the thematic variability identified in the analyzed publications. However, the scientific community seems to have been directing its research efforts toward issues related to the improvement of physical symptoms and the psychological well-being in patients with oncological pathologies. Furthermore, there are numerous studies focused on the cognitive behavioral intervention of patients with psychiatric diseases such as schizophrenia or degenerative diseases such as dementias, especially Alzheimer’s. Finally, drawing, painting and photography are the most recurrent visual arts modalities.

## Strengths and Limitations

The only information used in the study was retrieved from the core WoS collection. As a quantitative approach was the fundamental approach used in the development of the study, this could lead to a certain bias in the analysis of the results since the qualitative component of the publications in this research area was not taken into account.

The bibliometric approach is a good methodology to map the scientific development of the domain in terms of findings and gaps in research. A systematic review would be necessary to detail the available scientific evidence on the efficacy-effectiveness and efficiency of this type of intervention.

To the best of the authors’ knowledge, this is the first study to describe the evolution of the domain through the development of its lines of research.

## Conclusion

This work did not find the previous existence of any bibliometric analysis on the international scientific production in art therapy. The present study shows that the number of publications on the subject has multiplied substantially over the last decade. The results here are similar to those obtained in a similar study (Li et al., 2021) which evaluated the growth of music therapy research over the last 20 years.

The bibliographic data retrieved from the databases of the core WoS collection are analyzed by applying this retrospective, exploratory, descriptive and cross-sectional study, with a bibliometric approach.

In the 63 years between 1958 and April 2021, a total of 563 works on art therapy in the visual arts were found, indexed in the databases selected for the sample of this study, with psychology and rehabilitation being the main domains research.

In addition, the ability of art to broaden personal horizons means that these disciplines are able to transcend the individual aspects present in the disease (Marxen, 2011), bestowing on artistic therapies a polyvalent, multifaceted, multidisciplinary and conceivable character regardless of the application environment, the means and objectives of intervention, as described in this work.

In sum, through the proposed analysis, the authors conclude that, despite verifying a substantial growth in the number of publications on the subject during the last decade, the interest of researchers in visual arts as therapy continues to grow and progress.

The findings may provide useful information for art therapy (visual arts) researchers to identify new research directions and topics.

## Data Availability Statement

The datasets analyzed during the current study are available from the corresponding author on reasonable request.

## Author Contributions

All authors certify that they have participated sufficiently in the work to take public responsibility for the content, including participation in the concept, design, analysis, writing, or revision of the manuscript. LC-P and NR: conception and design of the study, acquisition of data and analysis, and interpretation of data. NR, MN, and JR: drafting the manuscript. MN and JR: revising the manuscript critically for important intellectual content. LC-P, NR, MN, and JR: approval of the version of the manuscript to be published.

## Conflict of Interest

The authors declare that the research was conducted in the absence of any commercial or financial relationships that could be construed as a potential conflict of interest.

## Publisher’s Note

All claims expressed in this article are solely those of the authors and do not necessarily represent those of their affiliated organizations, or those of the publisher, the editors and the reviewers. Any product that may be evaluated in this article, or claim that may be made by its manufacturer, is not guaranteed or endorsed by the publisher.

## References

[B1] Akhavan Tafti M. Rajabpour Azizi Z. Mohamadzadeh S. (2021). A comparison of the diagnostic power of FEATS and bender-gestalt test in identifying the problems of students with and without specific learning disorders. *Arts Psychother.* 73:101760. 10.1016/J.AIP.2021.101760

[B2] American Art Therapy Association (2021). *American Art Therapy Association.* Available online at: https://arttherapy.org/ (Accessed September 24, 2021).

[B3] Archambault É Campbell D. Gingras Y. Larivière V. (2009). Comparing bibliometric statistics obtained from the web of science and scopus. *J. Am. Soc. Inf. Sci. Technol.* 60 1320-1326. 10.1002/asi.21062

[B4] Asociación Profesional Española de Arteterapeutas. (2019). Qué Es Arteterapia? – Ate Arteterapia. Available online at: https://arteterapia.org.es/que-es-arteterapia/ (accessed August 22, 2021).

[B5] Bar-Sela G. Atid L. Danos S. Gabay N. Epelbaum R. (2007). Art therapy improved depression and influenced fatigue levels in cancer patients on chemotherapy. *Psychooncology* 16 980-984. 10.1002/pon.1175 17351987

[B6] Butcher H. Bulechek G. Dochterman J. Wagner C. M. (2018). *Clasificación de Intervenciones de Enfermería*, (NIC) 7 Edición. Barcelona: Elsevier. Available online at: https://www.elsevier.com/books/clasificacion-de-intervenciones-de-enfermeria-nic/butcher/978-84-9113-404-6

[B7] Capilla-Díaz C. Moya-Muñoz N. Matas-Terrón J. M. Pérez-Morente M. Á Álvarez-Serrano M. A. Montoya-Juárez R. (2020). Bibliometric analysis of qualitative research on patients’ experiences of intestinal stoma published between 2002 - 2018. *J. Adv. Nurs.* 76 1182-1191. 10.1111/jan.14321 32026509

[B8] Cheng P. Xu L. Zhang J. Liu W. Zhu J. (2021). Role of arts therapy in patients with breast and gynecological cancers: a systematic review and meta-analysis. *J. Palliat. Med.* 24 443-452. 10.1089/jpm.2020.0468 33507828

[B9] Cohen B. M. Hammer J. S. Singer S. (1988). The diagnostic drawing series: a systematic approach to art therapy evaluation and research. *Arts Psychother.* 15 11–21. 10.1016/0197-4556(88)90048-2

[B10] Darewych O. (2013). Building bridges with institutionalized orphans in Ukraine: an art therapy pilot study. *Arts Psychother.* 40 85-93. 10.1016/j.aip.2012.10.001

[B11] Goldner L. Lev-Wiesel R. Binson B. (2021). Perceptions of child abuse as manifested in drawings and narratives by children and adolescents. *Front. Psychol.* 11:562972. 10.3389/fpsyg.2020.562972 33519578 PMC7840510

[B12] Eum Y. Yim J. (2015). Literature and art therapy in post-stroke psychological disorders. *Tohoku J. Exp. Med.* 235 17-23. 10.1620/tjem.235.17 25744067

[B13] Favara-Scacco C. Smirne G. Schilirò G. Di Cataldo A. (2001). Art therapy as support for children with leukemia during painful procedures. *Med. Pediatr. Oncol.* 36 474-480. 10.1002/mpo.1112 11260571

[B14] Frohmann L. (2005). The framing safety project. *Violence Against Women* 11 1396-1419. 10.1177/1077801205280271 16204731

[B15] Gacto M. Gacto M. L. (2012). “Teaching art as a therapeutic method in psychomotorrehabilitation,” in *Proceedings of the 6th International Technology, Education and Development Conference*, Valencia 6683–6687.

[B16] Galletly C. Castle D. Dark F. Humberstone V. Jablensky A. Killackey E. (2016). Royal Australian and New Zealand college of psychiatrists clinical practice guidelines for the management of schizophrenia and related disorders. *Aust. N.Z. J. Psychiatry* 50 410-472. 10.1177/0004867416641195 27106681

[B17] Geue K. Goetze H. Buttstaedt M. Kleinert E. Richter D. Singer S. (2010). An overview of art therapy interventions for cancer patients and the results of research. *Complement. Ther. Med.* 18 160-170. 10.1016/j.ctim.2010.04.001 20688262

[B18] González M. I. D. González P. D. (2016). ¿Se ajustan las ventanas fijas de citación a las velocidades de maduración del impacto de las revistas científicas? *Investig. Bibliotecol. Archivonomía, Bibliotecología e Información* 30 73–89. 10.1016/j.ibbai.2016.02.004

[B19] Grenimann Bauch N. Bat Or M. (2021). Exploring paternal mentalization among fathers of toddlers through a clay-sculpting task. *Front. Psychol.* 12:518480. 10.3389/fpsyg.2021.518480 33737891 PMC7960673

[B20] Hass-Cohen N. Bokoch R. Goodman K. Conover K. J. (2021). Art therapy drawing protocols for chronic pain: quantitative results from a mixed method pilot study. *Arts Psychother.* 73:101749. 10.1016/J.AIP.2020.101749

[B21] Hays R. E. Lyons S. J. (1981). The bridge drawing: a projective technique for assessment in art therapy. *Arts Psychother.* 8 207-217. 10.1016/0197-4556(81)90033-2

[B22] Hughes E. G. da Silva A. M. (2011). A pilot study assessing art therapy as a mental health intervention for subfertile women. *Hum. Reprod.* 26 611-615. 10.1093/humrep/deq385 21247921

[B23] Jang S. H. Lee J. H. Lee H. J. Lee S. Y. (2018). Effects of mindfulness-based art therapy on psychological symptoms in patients with coronary artery disease. *J. Korean Med. Sci.* 33:e88. 10.3346/jkms.2018.33.e88 29542299 PMC5852419

[B24] Jensen A. Bonde L. O. (2018). The use of arts interventions for mental health and wellbeing in health settings. *Perspect. Public Health* 138 209–214. 10.1177/1757913918772602 29708025

[B25] Kim S. Han J. Kim Y. H. Oh Y. J. (2011). A computer art therapy system for kinetic family drawing (CATS_KFD). *Arts Psychother.* 38 17-28. 10.1016/j.aip.2010.10.002

[B26] Li K. Weng L. Wang X. (2021). The state of music therapy studies in the past 20 years: a bibliometric analysis. *Front. Psychol.* 12:697726. 10.3389/fpsyg.2021.697726 34177744 PMC8222602

[B27] Marxen M. E. (2011). Pain and knowledge: artistic expression and the transformation of pain. *Arts Psychother.* 38 239-246. 10.1016/j.aip.2011.07.003

[B28] Ozdemir L. Akdemir N. (2009). Effects of multisensory stimulation on cognition, depression and anxiety levels of mildly-affected alzheimer’s patients. *J. Neurol. Sci.* 283 211-213. 10.1016/j.jns.2009.02.367 19289242

[B29] Parr H. (2006). Mental health, the arts and belongings. *Trans. Inst. Br. Geogr.* 31 150-166. 10.1111/j.1475-5661.2006.00207.x

[B30] Potts L. R. (1958). Two picture series showing emotional changes during art therapy. *Int. J. Group Psychother.* 8 383-394. 10.1080/00207284.1958.11642587

[B31] Qué es Arteterapia? – ATe Arteterapia (2021). *ATe Arteterapia – Asociación Profesional Española de Arteterapeutas.* Available online at: https://arteterapia.org.es/que-es-arteterapia/ (Accessed September 24, 2021).

[B32] Quinn N. Hensey O. McDowell D. T. (2013). A historical perspective of pediatric publications: a bibliometric analysis. *Pediatrics* 132 406-412. 10.1542/peds.2013-0283 23979085

[B33] Riley R. G. Manias E. (2004). The uses of photography in clinical nursing practice and research: a literature review. *J. Adv. Nurs.* 48 397-405. 10.1111/j.1365-2648.2004.03208.x 15500534

[B34] Robertson B. (2001). Drawing a blank: art therapy for adolescent adoptees. *Am. J. Art Therapy* 39 74–79.

[B35] Rodríguez Gutiérrez J. K. Gómez Velasco N. Y. (2017). Redes de coautoría como herramienta de evaluación de la producción científica de los grupos de investigación. *Revista General de Información y Documentación* 27 279-297. 10.5209/RGID.58204

[B36] Sall J. Brenner L. Millikan Bell A. M. Colston M. J. (2019). Assessment and management of patients at risk for suicide: synopsis of the 2019 U.S. department of veterans affairs and U.S. department of defense clinical practice guidelines. *Ann. Intern. Med.* 171 343–353. 10.7326/M19-0687 31450237

[B37] Sarid O. Cwikel J. Czamanski-Cohen J. Huss E. (2017). Treating women with perinatal mood and anxiety disorders (PMADs) with a hybrid cognitive behavioural and art therapy treatment (CB-ART). *Arch. Womens Ment. Health* 20 229-231. 10.1007/s00737-016-0668-7 27645306

[B38] Scottish Intercollegiate Guidelines Network (2016). *Assessment, Diagnosis and Interventions for Autism Spectrum Disorders* Available online at: https://www.sign.ac.uk/media/1081/sign145.pdf

[B39] Stuckey H. L. Nobel J. (2010). The connection between art, healing, and public health: a review of current literature. *Am. J. Public Health* 100 254–263. 10.2105/AJPH 20019311 PMC2804629

[B40] Swan-Foster N. Foster S. Dorsey A. (2003). The use of the human figure drawing with pregnant women. *J. Reprod. Infant Psychol.* 21 293-307. 10.1080/02646830310001622105

[B41] Talwar S. (2007). Accessing traumatic memory through art making: an art therapy trauma protocol (ATTP). *Arts Psychother.* 34 22–35. 10.1016/J.AIP.2006.09.001

[B42] Tang Y. Li L. Yang Q. Shao Q. Xu Q. Shi H. (2021). Art therapy alleviates the levels of depression and blood glucose in diabetic patients: a systematic review and meta-analysis. *Front. Psychol.* 12:639626. 10.3389/fpsyg.2021.639626 33776864 PMC7994617

[B43] Wallace J. Yorgin P. D. Carolan R. Moore H. Sanchez J. Belson A. (2004). The use of art therapy to detect depression and post-traumatic stress disorder in pediatric and young adult renal transplant recipients. *Pediatr. Transplant.* 8 52-59. 10.1046/j.1397-3142.2003.00124.x 15009841

[B44] Wood M. J. M. Molassiotis A. Payne S. (2011). What research evidence is there for the use of art therapy in the management of symptoms in adults with cancer? a systematic review. *Psychooncology* 20 135-145. 10.1002/pon.1722 20878827

